# Advances in Nasal Biopharmaceutics to Support Product Development and Therapeutic Needs

**DOI:** 10.3390/pharmaceutics17101321

**Published:** 2025-10-11

**Authors:** Ben Forbes, Lucy Goodacre, Alison B. Lansley, Andrew R. Martin, Helen Palmer, Claire Patterson, Chris Roe, Regina Scherließ

**Affiliations:** 1 Institute of Pharmaceutical Science, King’s College London, London SE1 9NH, UK; lucy.goodacre@kcl.ac.uk; 2 School of Applied Sciences, University of Brighton, Brighton BN2 4GJ, UK; a.lansley@brighton.ac.uk; 3 Mechanical Engineering and Biomedical Engineering, University of Alberta, Edmonton, AB T6G 1H9, Canada; armartin@ualberta.ca; 4 Labcorp, Early Development Laboratories Ltd., Huntingdon PE28 4HS, UK; helen.palmer@labcorp.com; 5 Seda Pharmaceutical Development Services, Stockport SK8 3GX, UK; claire.patterson@sedapds.com; 6 Quotient Sciences, Nottingham NG11 6JS, UK; chris.roe@quotientsciences.com; 7 Department of Pharmaceutics and Biopharmaceutics, Kiel University, 24118 Kiel, Germany; rscherliess@pharmazie.uni-kiel.de

**Keywords:** nasal drug products, PBBM, nasal deposition, cell and mucus models, preclinical development, gamma scintigraphy

## Abstract

**Background/Objectives:** Nasal biopharmaceutics is the scientific understanding of product and patient factors that determine the rate and extent of drug exposure following nasal administration. The authors considered whether current biopharmaceutics tools are fit for the current and future needs of nasal product development and regulation. **Methods:** The limitations of current methods were critically assessed, unmet needs were highlighted, and key questions were posed to guide future directions in biopharmaceutics research. **Results:** The emergence of physiologically based biopharmaceutics models for nasal delivery has the potential to drive the scientific understanding of nasal delivery. Simulations can guide formulation and device development, inform dose selection and generate mechanistic insights. Developments in modeling need to be complemented by advances in experimental systems, including the use of realistic or idealized nasal casts to estimate the regional deposition of nasal sprays and refined in vitro cell culture models to study nasal drug absorption and the influence of mucus. Similarly, improvements are needed to address the practicalities of using animals in non-clinical studies of nasal drug delivery, and greater clinical use of gamma scintigraphy/magnetic resonance imaging is recommended to measure the delivery and nasal retention of different formulations in humans. **Conclusions:** Nasal drug delivery is a rapidly growing field and requires advances in nasal biopharmaceutics to support product innovation. Key needs are (i) validated clinically relevant critical product attributes for product performance and (ii) established links between how patients administer the product and where in the nose it deposits and dissolves in order to act or be absorbed, leading to its desired clinical effect.

## 1. Introduction to Nasal Drug Products

Nasal drug delivery is commonly used for the local treatment of diseases such as allergic rhinitis but is also well established for delivering drugs like desmopressin or calcitonin to the systemic circulation. The accessibility of the route and rapid absorption also make it attractive to deliver drugs to the systemic circulation for the treatment of emergency conditions such as opioid overdose, severe hypoglycemia and acute anaphylaxis [1,2,3], where it offers a rapid onset of action while avoiding the disadvantages of the parenteral route. Furthermore, the nasal route can be used to deliver vaccines to the nose-associated lymphoid tissue (NALT) against a variety of infections (not just respiratory), e.g., SARS-CoV-2 [4]. In addition, the nasal cavity provides a selective route of drug delivery to the brain [5] providing an important opportunity to treat central nervous system (CNS) diseases such as dementia.

The global nasal drug and vaccine delivery market is predicted to expand at a Compound Annual Growth Rate (CAGR) of 6.6% from 2025 to 2030 reaching a market size of USD 101 billion [6]. The Asia Pacific market is projected to grow at the fastest rate in the upcoming period, while North America currently holds the largest market share at 30% [7]. Driving this is the increasing demand for needle-free methods of administration and advances in delivery technology for the new era of biologics, which has increased the range of treatable conditions. The nasal route also supports the growing move to self-administration and home care, and the increase in the aging population has stimulated a demand for better drug delivery to the brain to address the attendant rise in neurological conditions [8].

### 1.1. Compendial and Regulatory Requirements

Nasal products include nasal drops, mostly used for infants, nasal sprays, either solution or suspension, and nasal powders. Each product can be characterized for various aspects depending on the needs and concerns [9]. However, the compendial requirements remain quite vague. The European Pharmacopoeia in its monograph “Ph. Eur. 11.0/0676: Nasal Preparations” requires uniformity of the delivered dose and number of deliveries, along with other tests for multidose preparations, which are the most common products on the market. Determination of the delivered dose, however, is not as simple as it sounds, as it requires a dose collection apparatus that is able to reproducibly capture the full dose, without spillage or loss, after the actuation of the spray in a manner as described in the patient information leaflet. This often means upright spraying giving rise to dripping from the dose collecting vessel, which needs to be placed upside down. The advice of the Ph. Eur. to use the dose collection apparatus as described for inhalation products is not very helpful (and will be replaced in future versions) in this context, as it neglects important requirements such as a ventilation opening and air bypass, upright orientation and the fact that a nasal spray dose typically is liquid and of a larger volume than an inhaled dose. Thus, scientists use various non-standardized devices to collect the nasal spray dose.

Further requirements for nasal products can be found in the U.S. Food and Drug Administration (FDA) Guidance for Industry on Nasal Spray and Inhalation Solution, Suspension, and Spray Drug Products-Chemistry, Manufacturing, and Controls Documentation [10] and the corresponding European Medicines Agency (EMA) guideline on the pharmaceutical quality of inhalation and nasal products [11]. Apart from some general characteristics of nasal products, such as pH, osmolarity and viscosity, the FDA focuses on spray performance (e.g., content uniformity, plume geometry and spray pattern). It is known that different spray devices may create different spray plumes, which can result in differences in therapeutic effect [12]. This is probably a consequence of differences in intranasal deposition. However, the mere determination of plume geometry, spray angle and pattern are probably not meaningful to predict regional deposition. To get a better idea of regional deposition, various physical [13,14,15,16] and in silico [17,18] nasal cast models are in use, with individual advantages and drawbacks. As new techniques are introduced to support Quality-by-Design (QbD)-driven development of nasal products, thorough validation across models using different nasal product types will be required to justify their use.

Another aspect of nasal delivery concerns the amount of material potentially being inhaled into the lungs, as this may cause side effects and could pose a potential safety risk. Consequently, both the FDA and EMA request the determination of droplet size distribution, with an emphasis on the percentage of droplets below 10 µm. It is assumed that these could exit the nose and get inhaled into the lower respiratory tract, and this should be minimized. While the FDA suggests using laser diffraction for the determination of the volume of droplets below 10 µm, the new EMA guideline mentions (abbreviated) cascade impactor methods as an option if the product is not a solution. A current work order of the European Directorate for the Quality of Medicines and HealthCare (EDQM) addresses this, and a method “for the determination of the mass fraction below 10 µm in nasal sprays” is to be established. Given the limitations of laser diffraction in the assessment of inhalation products (in terms of aerodynamic particle size and the distinction between drug and drug-free particles) [19], the use of an impactor method for the determination of the mass fraction below 10 µm will be required if general applicability to all nasal sprays is the aim.

With this requirement in mind, the University of Kiel evaluated different impactor setups for determining mass fraction below 10 µm, namely the full Next-Generation Pharmaceutical Impactor (NGI), an abbreviated NGI (rNGI) and the Fast Screening Impactor (FSI) [20,21]. All three impactors typically use the USP throat as an inlet, which cannot be used directly with a nasal spray due to its orientation (the USP throat has a horizontal opening, while a nasal spray is operated vertically). Thus, the metal inlet port first described by Williams et al. [22] and the 1 L glass expansion chamber were evaluated as alternative setups.

**Figure 1:**
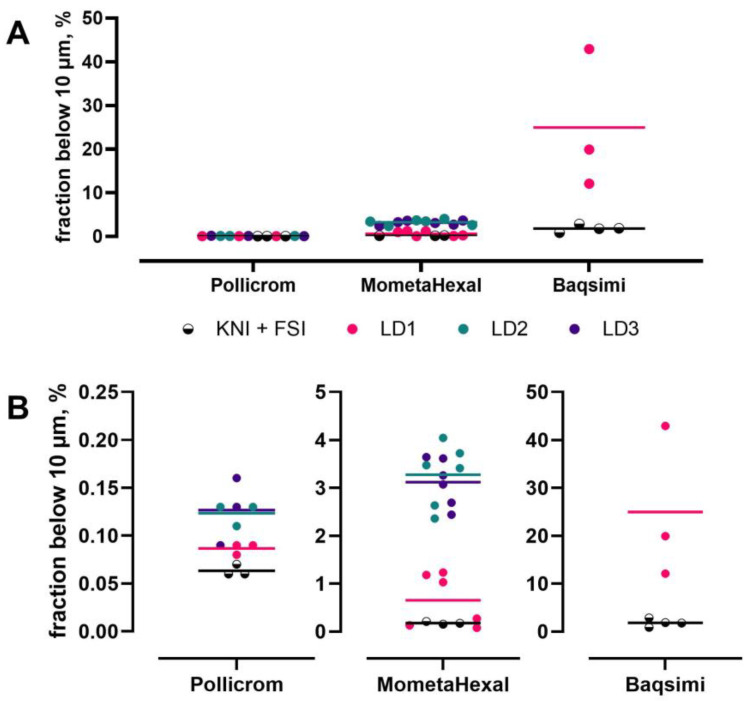
Comparison of the fraction below 10 µm of a nasal solution (Pollicrom), a nasal suspension (MometaHexal) and a nasal powder (Baqsimi) product determined by the aerodynamic setup (KNI + FSI) and laser diffraction (LD1, LD2 and LD3 are different batches of the respective product). (**A**) shows an overview, and (**B**) depicts the relevant parts magnified (different y-axes). Points are individual data; bars show the mean. Figure reproduced from [23] with permission.

Using a commercial nasal spray solution and an airflow of 30 L/min, many stages of the full NGI were not needed for the distinction between above and below 10 µm and led to problems with sample loss and the limit of quantification. The rNGI and FSI performed much better, with the FSI being easiest in handling. Overall, the mass fraction below 10 µm was very small (much less than 1%) for nasal solution sprays, which is a further challenge for the method. Using the FSI, both inlets gave comparable mass fractions below 10 µm (difference was non-significant). The metal inlet resulted in higher variability in the results, which was probably due to liquid spill from the inlet port to other compartments. Furthermore, the metal inlet port could only be used at a fixed angle of 60°, which was contrary to the patient information for use of the product. For these reasons, the metal inlet port was not favored. The glass expansion chamber enabled full expansion of the spray cloud, which could result in an overestimation of the mass fraction below 10 µm due to droplet evaporation. These limitations led to the development of a bespoke nasal inlet for an impactor that is compatible with the existing USP throat: the Kiel Nasal Inlet (KNI) [21,23]. As well as solution sprays, the KNI can also be used for nasal suspension and nasal powders [24]. Especially for the latter, the differences between laser diffraction assessment and cascade impaction are significant and underline the importance of using an impaction method over particle size measurement (Figure 1).

### 1.2. Nasal Drug Product Bioequivalence

Both the European Medicines Agency (EMA) and the U.S. Food and Drug Administration (FDA) rely on a comprehensive set of in vitro characterization methods to establish bioequivalence for nasal products, particularly for locally acting drugs where systemic absorption is not the primary measure of availability. While the specific guidance documents differ in detail, both regulatory bodies emphasize a “weight-of-evidence” approach that combines various in vitro tests to demonstrate equivalence between a generic and reference product. The key tests required in both regions include delivered dose uniformity throughout the product’s life, from beginning to end, spray pattern and plume geometry to confirm that the physical characteristics of the spray are comparable and droplet size distribution to ensure the drug reaches the correct site of action within the nasal cavity. For BE, an aerodynamic mass fraction < 10 µm provides an important safety measure, and limits compared to the reference product may be introduced in the future.

### 1.3. Nasal Biopharmaceutics

Biopharmaceutics adds an extra dimension to drug delivery science by interfacing the drug product with physiological realities. Current quality methods fail to account for the actual use of a nasal product, and this review summarizes current and emerging methods used to study nasal biopharmaceutics and predict the clinical performance of nasal products. A biopharmaceutics framework for nasal delivery needs to encompass deposition in the nasal cavity, followed by the dynamics of absorptive and non-absorptive clearance, and requires in vitro tests that are in vivo predictive.

## 2. Physiologically Based Biopharmaceutics Modeling (PBBM)

**Figure 2:**
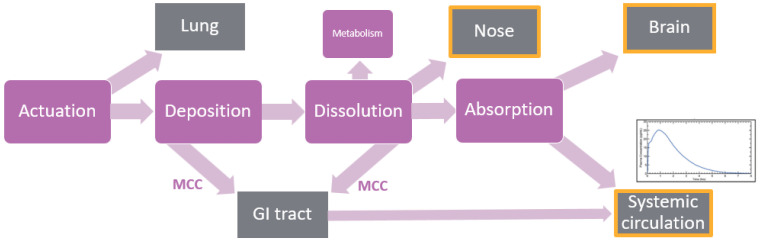
An illustrative physiologically based biopharmaceutics model for nasal drug delivery. MCC = mucociliary clearance.

PBBM is a specific subset of in silico physiologically based pharmacokinetic (PBPK) modeling that focuses on understanding the impact of physiology, the drug and formulation on drug concentrations at the target site of action. It integrates drug metabolism and pharmacokinetics (DMPK)-based models of traditional PBPK software with physical chemistry models of dissolution and other relevant processes (Figure 2). PBBM is used extensively during product development for oral drugs to inform compound selection, formulation design and dose prediction and is being used increasingly in regulatory settings to build a “safe space” to gain regulatory flexibility [25]. There is great potential for PBBM to be similarly impactful in nasal drug delivery. For instance, PBBM could help identify rate-limiting steps in the nasal absorption of drugs intended for systemic delivery (for example, highly potent peptides unsuitable for oral administration, where a non-invasive, needle-free formulation is desirable). It may highlight the need for solubility enhancement, permeation enhancers, mucoadhesive excipients or combinations thereof or perhaps inform whether batch-to-batch differences in particle size might affect the local concentration/bioequivalence of a nasal suspension intended for topical action. With growing insights into nose-to-brain pathways, PBBM may also enable more accurate predictions of brain concentration profiles and support dose selection for CNS-targeted therapies.

To build PBBM models for nasal delivery, species-specific physiological parameters must be combined with measured or predicted drug and formulation attributes to create dynamic models simulating the absorption, distribution, metabolism and excretion (ADME) behavior of the drug. These models rely on mathematical descriptors linked by differential equations to predict concentration–time profiles at the target site, whether in the nasal cavity, systemic circulation or brain. When constructing a PBBM model for nasal delivery, several inter-related physiological and formulation-dependent processes must be considered. The journey begins with actuation of the device where user technique, device design, and formulation characteristics collectively influence the spray plume geometry, droplet or particle size distribution and the resulting deposition pattern within the nasal cavity. Particles smaller than approximately 10 µm are more likely to be inhaled into the lungs, whereas excessively large droplets may drip from the nostrils or be swallowed post-nasally. The preferred site of deposition is dictated by the intended therapeutic target, whether local, systemic or central nervous system (CNS). Following deposition, any solid particles will need to dissolve in the low-volume nasal secretions and subsequently diffuse across the mucus layer before being absorbed. Mucociliary clearance and local metabolizing enzymes will be working to clear the formulation from the nose. The enzymatic barrier of the nasal mucosa creates a pseudo alternative first-pass effect. It is well documented that cytochrome P-450 activity in the olfactory region of the nasal epithelium is high [26], and Phase II activity has also been found in the nasal epithelium [27]. The delivery of peptides and proteins may also be hindered by the peptidase and protease activities in the nasal mucosa [28].

Importantly, it may not be necessary to mechanistically simulate every process. A pragmatic approach focusing on rate-limiting steps is often more feasible and informative.

Further work is required to develop in vitro methods for characterizing critical input parameters for nasal PBBM. For instance, a volume-restricted Transwell-type dissolution setup may be more suitable than a bulk USP I/II dissolution apparatus for generating meaningful, biorelevant dissolution profiles. For oral delivery, we learn a great deal from absorption behavior in preclinical species, where gastrointestinal tract parameters are well characterized and can be scaled according to species. However, nasal geometries vary widely across species, making it very difficult to ensure dosing in rodents, for example, that is representative for scaling to human, as discussed in further detail in Section 5. Some commercial software platforms are beginning to introduce nasal modeling functionality into their programs. GastroPlus^®^, for example, contains a pulmonary/intranasal absorption model in its Additional Dosage Routes Module (ADRM); however, we found no peer reviewed publications regarding its functionality or performance. We invite users to publish their findings, including validation datasets and performance metrics such as prediction error bands for the C_max_, AUC and brain/plasma ratios. Gonda (1998) published a pseudo compartmental model for nasal delivery comprising a series of linear differential equations: a simplified model that employed a user-defined input parameter for percent dose deposition in the anterior region of the nasal cavity [29]. Others have published more complex hybrid computational fluid dynamics (CFD)–PBPK models, whereby particle deposition data from a 3D model is mapped onto an interior surface model, and then CFD simulations account for particle movement due to mucociliary clearance and particle dissolution, diffusion and absorption [30]. A compartmental PK model then generates the PK profile. Some such programs have received FDA funding [31], reflecting growing regulatory interest.

The authors advocate for broader adoption of PBBM in nasal delivery and urge the scientific community to collectively rise to the challenge of building upon existing models. This includes generating and sharing relevant parameters and datasets, advancing mechanistic understanding (particularly in nose-to-brain delivery) and validating models with experimental data to assess their predictive performance. Such efforts will foster a cycle of continuous improvement, ultimately enhancing the utility of PBBM in nasal drug development. Examples of model input parameters for the main processes described in a typical PBBM (Figure 2) are listed in Table 1. Further consideration of these parameters is given in the subsequent sections of the manuscript.

## 3. Modeling Deposition in the Nasal Cavity

Nasal drug products are combination products, with interaction between the formulation and device influencing their performance. The vast majority of marketed nasal drug products are aqueous solutions or suspensions, delivered using nasal spray pumps. However, propellant-based and powder-based products have also been developed, along with novel delivery devices that may offer advantages over traditional spray pumps, for example, in their ability to target delivery to regions of interest within the nasal airways.

Standardized in vitro tests for nasal sprays are well established [10]. Several of these tests, including measurements of spray content uniformity, spray pattern and plume geometry and droplet size distribution, are particularly useful for inferring the reproducibility of dosing to the nasal airways and in identifying similarities or differences between products, including test versus reference products. Presumably, two nasal spray products that reproducibly deliver similar emitted doses, with similar spray pattern, plume geometry and droplet size distributions, will produce similar patterns of deposition within the nasal airways. However, established in vitro tests do not go so far as to quantify predicted regional doses. Furthermore, while these tests have been developed for nasal sprays, their applicability to other nasal dosage forms, including nasal powders, is uncertain.

**Figure 3:**
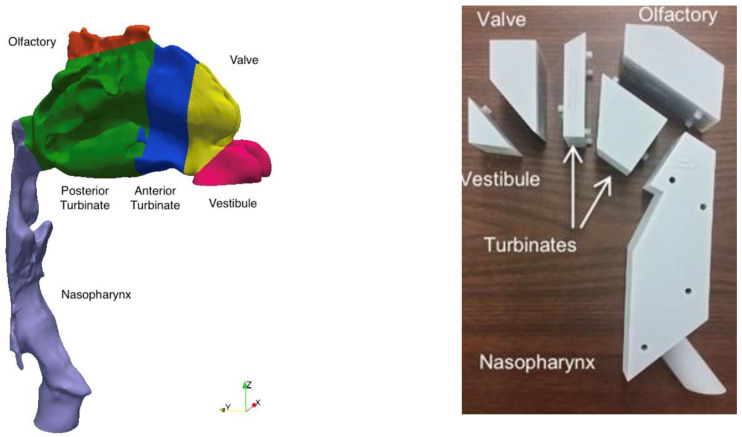
Comparison of in silico (**left**) vs. experimental (**right**) models for predicting regional drug deposition in the nasal cavity. Left image reproduced from Kiaee et al., 2018 [17], with permission from John Wiley and Sons.

Considering these limitations, a number of groups have investigated regional deposition of nasal drug products in realistic nasal geometries derived from medical images. Both experimental (in vitro) [15,32,33,34,35,36] and numerical (in silico) [17,37,38,39] approaches have been employed, which offer complementary information (Figure 3). Whereas in vitro experiments are typically limited in scope by the availability of commercial, or prototype, drug–device combinations to test, in silico studies may be conducted over much wider parameter ranges, limited only by available computational resources and the range of validity of assumptions inherent to a given numerical model. Similarly, where in vitro experiments commonly evaluate regional deposition over discrete regions of interest, often using sectioned physical models that may be disassembled into constituent regions for assay [15,32,34], in silico simulations map particle motion and deposition continuously throughout the nasal geometry. Nevertheless, in vitro experiments are well suited to the characterization and comparison of existing, or prototype, nasal products, as these products are used directly in the tests performed. In contrast, when conducting in silico studies, it is challenging to fully define the initial and boundary conditions for specific products (including the size distribution of droplets or particles as they enter the nose, along with the distribution of their velocities, in both magnitude and direction).

Collectively, studies investigating regional deposition of nasal drug products in realistic geometries have highlighted considerable inter-subject variability in deposition patterns [15,17,33,35]. Deposition in specific regions of interest is not easily correlated with any single airway dimension or length scale. For example, Hosseini et al. [36] observed that posterior nasal spray deposition measured in vitro in a set of 20 realistic nasal airway replicas was influenced by at least ten independent airway dimensions and administration parameters. This renders a priori prediction of regional nasal deposition based, e.g., on correlations incorporating patient characteristics and anticipated administration/use parameters challenging, such that further use of in vitro and in silico methods incorporating nasal geometries to estimate regional deposition profiles is warranted [36,40].

Given the variability in regional deposition between different nasal geometries, the selection of a single geometry, or limited set of geometries, for use in regional deposition studies requires careful consideration and should be validated, as much as possible, against in vivo data [16,41]. To this end, Alfaifi et al. [42] identified three realistic geometries selected based on their tendency to exhibit low, medium or high values of posterior nasal spray deposition relative to deposition evaluated in vitro in a larger set of realistic geometries. Posterior deposition was measured in vitro using the three geometries for a commercial mometasone furoate nasal spray and found to be in reasonable agreement with previously published in vivo data for the same nasal spray product, with some discrepancy attributed to differences in the definition of the posterior nasal airway region between the in vitro and in vivo studies [42]. Using an alternative approach, Kiaee et al. [43] proposed an idealized nasal airway designed to mimic regional nasal deposition data in realistic geometries obtained by conducting extensive in silico simulations over a wide-ranging parameter space [43]. Subsequently, Chen et al. [15] evaluated regional deposition of a commercial cromolyn sodium nasal spray measured in vitro in a physical prototype of the idealized geometry, consisting of anterior, turbinate, olfactory and nasopharynx regions. Regional deposition measured in the idealized geometry was found to consistently match average regional deposition measured in a set of realistic geometries. In a further study, Chen et al. [44] demonstrated that in vitro experiments conducted using a commercially available metal version of the idealized geometry, the Alberta Idealized Nasal Inlet, or AINI (Copley Scientific), were in good agreement with previously published in vivo data describing regional deposition of three distinct nasal drug products: an aqueous solution nasal spray, an aqueous suspension nasal spray and a pressurized metered-dose nasal spray device. Subsequently, several groups have reported in vitro methods using the AINI to evaluate regional deposition of nasal powders [45,46,47]. In this case, coating the inner surfaces of the AINI with a layer of liquid, mimicking the mucus layer that lines the nasal airway in vivo, is critical to avoid excessive particle bounce [45]. To date, no study has directly compared in vitro deposition data from the AINI model with in vivo medical imaging data to establish an in vivo–in vitro correlation (IVIVC) for nasal powders.

## 4. In Vitro Biological Models for Nasal Biopharmaceutics

### 4.1. What Regions of the Nasal Cavity Do We Want to Model?

When considering the delivery of drugs to the nasal cavity, there are several different anatomical regions that are of interest depending on the desired effect of the product. For local and, particularly, systemic drug delivery, it is likely that the respiratory mucosa of the turbinates, with its relatively large surface area (approximately 150 cm^2^) for drug absorption, would be of most interest. In this region, mucus is secreted by goblet cells and submucosal glands [48]. For direct nose-to-brain delivery, it is the olfactory region of the nose (approximately 10 cm^2^) that is of most interest. In humans, patches of olfactory mucosa are interspersed with respiratory mucosa, and mucus is secreted by Bowman’s glands found in the lamina propria beneath the olfactory epithelium. Polymeric mucins are a key component of the mucus that lines the nasal cavity, giving it its gel-like properties. In mice, mucins 1, 5AC and 5B have been reported to have different patterns of expression in olfactory and respiratory epithelia [49,50], and the secretions might therefore be expected to behave differently. The delivery of vaccines would be best targeted to the nasopharynx, where the nasal-associated lymphoid tissue (Waldeyer’s ring (tonsils, adenoids and other lymphoid tissue)) is found. Mucus from the entire nasal cavity will be swept across this region by mucociliary clearance on its way to being swallowed.

**Figure 4:**
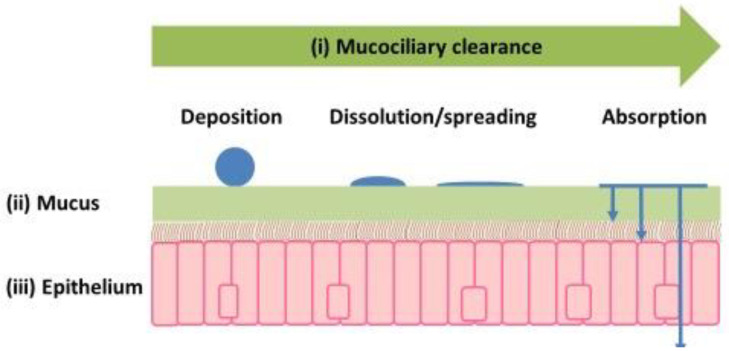
The fate of particles or droplets deposited in the nasal cavity.

Upon administration, the droplets (solutions or suspensions) or dry particles (powders) in nasal formulations will deposit on the mucus lining the nasal cavity (Figure 4). Droplets will spread, and drugs in solution will be immediately available for diffusion across the mucus and epithelium. For drugs in suspension and those delivered as powders, dissolution will be necessary before diffusion can occur. All of this occurs against a backdrop of mucociliary clearance, which limits the time available for these processes. The bulk of a solution deposited in the turbinate region of the nasal cavity is cleared in 20–30 min [51], and it has been suggested that the high density of cilia in the nasal cavity would also allow the mucociliary clearance of formulations deposited in areas with non-motile cilia such as the olfactory mucosa (olfactory cilia do not beat) [52].

When modeling the nasal epithelium, one can consider whether complete recapitulation of the native tissue is required or just key (rate-limiting) features. These features include (i) mucociliary clearance, which could be captured by the duration of the experiment, as many in vitro cell models do not have a functioning mucociliary clearance apparatus, as well as (ii) mucus and (iii) the epithelium itself (Figure 4).

### 4.2. How Important Is Mucus in Nasal Drug Delivery?

The role of mucus in drug delivery is often neglected. It plays a role in the dissolution of the drugs administered in suspension and powder form and in the dissolution of soluble excipients when the product is a powder. If dry polymers are included in the formulation to increase retention time, then mucus will play a role in the hydration of the polymer. The polymer will compete with the mucins for the available water [53], and the degree of hydration will affect the mucoadhesion of the polymer. Mucus is also able to provide a diffusion barrier to drugs and particles, and some drugs will bind to mucus. The secretion of mucus can also be used as a measure of biocompatibility if it is assumed that local irritancy/toxicity will increase the secretion of mucus. Certain excipients used in nasal products have been shown to increase mucin secretion [54].

A variety of different models have been used to study the role of mucus in biopharmaceutics and are considered in several reviews [55,56,57]. One is the use of mucus-secreting cells cultured on permeable supports at an air–liquid interface. In these, mucus is secreted by goblet cells, and there is no glandular contribution. There are several mucus-secreting cell lines that have been used to model the respiratory mucosa of the turbinates, e.g., human lung cell line (Calu-3) [58,59,60], rat tracheal cell line (SPOC-1) [61], human bronchial cell line (UNCN3T) [61], human airway epithelial cell line (NuLi-1) [62] and human nasal epithelial cell line (RPMI 2560) [63]. In addition, primary cultures of rat and human nasal/airway epithelia can be produced “in house” or are available commercially, e.g., MucilAir, EpiAirway, EpiNasal, etc., although these tend to be more variable than cell lines [64,65]. Cell models of the olfactory mucosa and nasopharynx are more complex, and there are only a few examples of their use [66,67]. An overview of different mucus-secreting cell models is demonstrated in Table 2.

### 4.3. How Best to Validate Existing and New In Vitro Cell Models

Any cell culture model needs to be validated to show that a good in vitro–in vivo correlation exists, preferably in humans rather than rodents. To minimize variability in the in vitro data, it can be argued that it would be preferable for each cell line to be cultured under well-defined, standardized culturing conditions and for a standard experimental protocol to be used for drug transport experiments. Furthermore, each model should be validated against a standard set of compounds with a range of physicochemical characteristics (e.g., molecular size and lipophilicity) to include substrates for known transporters and efflux pumps. To ensure that a cell model can secrete sufficient mucus to be useful, the permeation of a compound known to be affected by mucus, e.g., testosterone should be studied in the absence and presence of mucus. This can be achieved by depleting mucus by washing and comparing permeation across washed and unwashed cells. Alternatively, mucus secretion can be increased using a secretagogue such at ATP and permeation compared across treated and untreated cells. If a difference in the rate permeation of the model compound is observed, then the model can be used further to test the effect of mucus on the permeation of novel compounds [61].

## 5. Non-Clinical In Vivo Models for Nasal Drug Product Development

The similarities and differences in structure and architecture between the nasal cavities of man and the most commonly used animal models are well characterized and are presented in Table 3. Animals with a long snout (including rodents and dogs) generally have a more complex nasal architecture than man, with a greater surface area-to-overall volume ratio. Furthermore, the route to the nasopharynx is significantly more complicated, and the area of softest tissue between respiratory passages and brain is proportionally noticeably smaller [107]. This makes intranasal dosing efficacy and target exposure challenging to translate to the clinical situation, particularly for any type of quantitative translation of brain exposure.

Fortunately for regulatory purposes, the technical difficulties of dosing animals are mitigated by an understanding that although the intranasal model cannot necessarily translate effectively for exposure and deposition purposes, the model is still valid for assessment of local tolerance and systemic toxicity. Instillation rather than insufflation is generally considered sufficient, particularly for rodent species.

A great deal of the effort in the development of intranasal medicines for clinical use has been in the development of an effective administration device, appropriate particle size distribution aimed at upper respiratory deposition and minimal lung exposure and, thus, a consistent dose. Given the difference in size and anatomy between non-clinical animal models and humans, these efforts are generally not useful for the animal model. For optimal non-clinical exposure, a smaller particle size, lower pressure and longer administration of a lower formulation concentration would be necessary. Fortunately, the clinical formulation may still be used for non-clinical safety provided one is aware of the limitations in translation.

The greatest challenge is the administration of dry powder to rodent species. Appropriate devices are rare, albeit currently in development, and may not be able to offer mucosal coverage that represents that of a spray device in humans. It should be considered that inhalation administration of powders may offer a more consistent and reproducible dose for safety assessment in rodents, particularly where brain exposure is of particular interest.

In summary, animal models for safety assessment of intranasal pharmaceuticals are well characterized, particularly regarding suitability for safety and tolerability assessment, but due to relative anatomical complexity, local exposure and deposition data should be considered indicative rather than representative.

## 6. Clinical Tools in Nasal Drug Product Development

Scintigraphic imaging and nasal wick evaluations are examples of clinical development tools that can be used to understand the inter-relationship of nasal drug, dosage form and device performance characteristics, as well as the robustness, rate and extent of drug deposition, residency and absorption in vivo.

### 6.1. Gamma Scintigraphy

**Figure 5:**
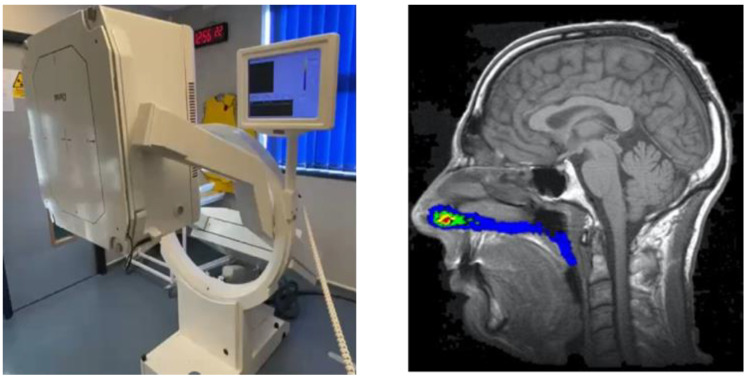
Gamma camera used to capture scintigraphic images (**left**) and composite (co-registered) MRI scan and nasal gamma scintigraphic image (**right**, colored areas represent scintigraphic radiation count intensity). Images from Quotient Sciences.

Two-dimensional, non-invasive gamma scintigraphy remains the most widely used imaging methodology to assess in vivo nasal drug delivery, although three-dimensional alternatives such as single-photon emission computed tomography (SPECT) and positron emission tomography (PET) are available [109]. Clinical studies using gamma scintigraphy allow for the quantification of deposition and clearance of formulations that have gamma-emitting radionuclides incorporated, typically technetium-99m (^99m^Tc, radioactive decay half-life of 6 h), and visualized through image acquisition with a gamma camera. For nasal imaging studies, an individual’s nasal cavity shape and regions are defined through magnetic resonance imaging (MRI) with the images from the gamma camera being subsequently aligned with the MRI images on an individual specific basis (Figure 5).

**Figure 6:**
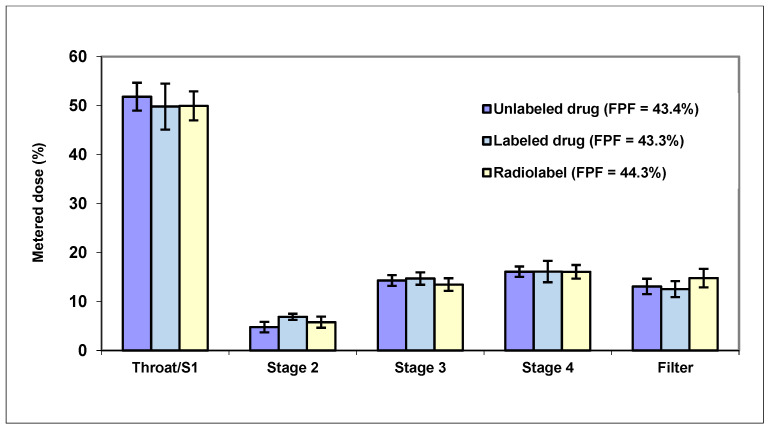
Validation impinger data (mean, n = 5) demonstrating no impact on the deposition of an orally inhaled drug product post-radiolabeling and that the radiolabel measured aligns with % drug indicating that it is an appropriate surrogate marker (FPF—fine particle fraction).

Deposition over time in the nasal cavity as a whole and/or regionally can then be quantified along with the amount of radiation being swallowed via the nasopharynx and reaching the lungs. In addition, images can capture the material remaining within the delivery device and any nasal wipes taken. Clinical studies using gamma scintigraphy in conjunction with MRI allow for key nasal delivery questions to be addressed, such as “how much is delivered?”, “what is the spread of deposition?”, “how long does it reside?” and “how much reaches the lungs?”. Where oral deposition or absorption is minimal following nasal administration, imaging data can also be considered in conjunction with any PK (blood, plasma and/or cerebrospinal fluid related) and biomarker data obtained from the same study subjects to provide greater insight into product performance. Importantly, the ^99m^Tc radiolabel is not chemically bound to the drug in these studies; instead, it acts as a surrogate marker within the product formulation and is chelated to diethylenetriaminepentaacetic acid (DTPA). The ^99m^Tc-DTPA complex itself is only absorbed by the nasal mucosa in minute quantities [110]. This radiolabel can be spiked into solution formulations and suspension vehicles for use in nasal devices including droppers, pump sprays, pressurized metered-dose inhalers (pMDIs), nebulizers and soft mist inhalers. Impactor, impinger or nasal cast methods [111] can be used to confirm that the process of radiolabeling itself does not affect product performance and that the surrogate marker is relevant, with pre- and post-labeling products tested and compared through both drug and radiolabel analyses of the fine particle fraction (FPF) % as a marker of the nasal product performance (Figure 6).

Whilst surface labeling of drug particles for suspensions or dry powder systems has been performed in the past, this is extremely challenging technically and is no longer in widespread use. This could be revisited, should the predicted rise in the nasal drug delivery market warrant it, and this in vivo information could support additional nasal biopharmaceutics understanding.

**Figure 7:**
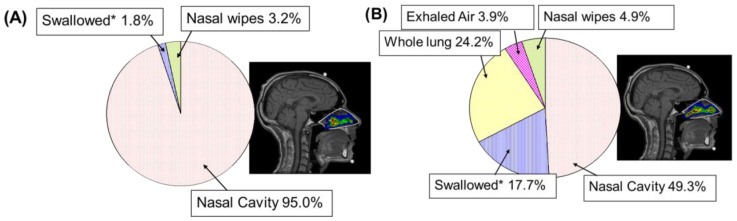
Clinical comparison of in vivo nasal delivery between (**A**) a conventional pump spray and (**B**) a nebulizer using gamma scintigraphy and MRI. * Swallowed material includes that deposited in the nasopharynx, oropharynx, oesophagus and stomach.

Clinical studies are useful for comparison of nasal delivery from different devices. For example, a comparison of a pump spray and a nebulizer found that the pump spray delivered the majority of the formulation to the nasal cavity whilst the nebulizer delivered more formulation to the lungs and more was swallowed, indicating that the pump spray was most suited for local nasal delivery (Figure 7).

Clinical imaging can also investigate the influence of patient variables. For example, the impact of different head positions on percentage olfactory regional deposition of radiolabeled placebo nasal drops has been examined, where the angle of the nose relative to the bed was adjusted between 40° to 90°. The highest olfactory deposition and least variability observed over time was achieved at the 90° angle [112].

### 6.2. Nasal Wicks

The clinical use of nasal wicks provides an opportunity to evaluate in vivo biomarkers. The use of nasal wicks offers increased the sensitivity and decreased the variability in monitoring clinical biomarker endpoints following nasal delivery in comparison to more traditional nasal washes [113]. In a typical example, nasal wick bioanalysis of cytokine levels was evaluated following 5 days of nasal dosing with a reference formulation and a novel test formulation designed to have better manufacturability and reduce the number of doses a patient would require. The change in specific cytokine levels from baseline results obtained through the nasal wicks showed that the new formulation was performing similarly to the reference formulation and demonstrated its potential to progress in development. Although some adverse events were attributed to the use of nasal wicks, these were considered minor.

These examples illustrate how nasal drug delivery assessment in vivo can be used to compare different delivery devices, investigate delivery to nasal regions in addition to residence and clearance and assess drug and biomarker levels in mucosal lining fluid. There are multiple clinical evaluation tools available that could be employed to support improving our understanding of nasal biopharmaceutics, and a suggested next step would be to agree which of these clinical methods provide the most relevant data to progress this area.

## 7. Conclusions

The aim of this review was to consider whether the methods and models for assessing nasal biopharmaceutics are fit for current and future needs. The current surge of interest in nasal drug delivery is driven by the success of new nasal products for emergency applications and systemic delivery, the potential of nasal delivery for brain targeting and as an alternative to parenteral delivery for the new generations of biologics. To achieve this potential, new and improved models and methods to study nasal biopharmaceutics are required. A vision for the future is the development of an evidence-based practice guide that ties the intended target (local/systemic/CNS) to a preferred deposition region, with recommended device/formulation levers supported by key tests and PBBM parameters.

The existing unmet needs, as perceived by the authors, are summarized in Table 4. These concern the nasal drug product itself and whether the methods provide adequate characterization for its use by the patient. Once administered, various methods exist (computational, in vitro and in vivo) to study drug availability at the deposition site, which may be critical to the clinical effectiveness of the product, e.g., if targeting the olfactory region is a priority, and questions arise regarding the best models and methods to quantify this. Methods and models are required to study droplet/particle impaction behavior, dissolution and absorption after deposition and the effect of mucus interactions and mucociliary clearance on these. The information generated by these models, along with in vivo/clinical data for validation, can inform the development of nasal PBBM. Biopharmaceutics modeling can enhance the mechanistic understanding of nasal delivery, similar to the way that the development of PBBM for oral inhalation [114] informed the development of the inhalation Biopharmaceutics Classification System [115] and has helped identify biopharmaceutics needs for lung delivery [116].

## Figures and Tables

**Table 1:** Methods for generating inputs for physiologically based biopharmaceutics models (Figure 2) and typical data ranges.

Model Input	Measurement Method	Typical Range/Units
Regional Deposition	In vitro nasal casts, gamma scintigraphy and computational fluid dynamic modeling	% of dose per region (e.g., anterior vs. posterior)
Dissolution/Spreading Under Volume-Limited Conditions	Simulated nasal fluid dissolution, e.g., Transwell systems	% dissolved with time (minutes to hours)
Mucus Thickness/Viscoelasticity	Imaging (confocal microscopy), histology, rheometry, microrheology	5–20 µm, 1–1000 cP
Absorption/Permeation	Caco-2, PAMPA, mucus-secreting airway cell models (e.g., Calu-3, SPOC-1, UNCN3T, NuLi-1, RPMI 2560, MucilAir and EpiNasal) and ex vivo nasal tissue	10^−6^ to 10^−3^ cm/s
Mucociliary Clearance Half-Life	Gamma scintigraphy and in vivo studies	15–30 min (varies by region)
Enzymatic Degradation (e. g., Esterases)	Biochemical assays and LC-MS/MS profiling	Half-life: minutes to hours; enzyme activity varies by region

**Table 2:** Mucus-secreting cell models of the nasal epithelium.

Epithelial Model	Source	Mucin Secretion	TEER Cells Cultured at ALI (Ω.cm^2^)	TJ Proteins	Transporter Functional Studies	Recommended Use Cases		
						Permeation	Toxicity	Enhancer Screening
**Primary Cells**	Various species including human, rat, sheep and pig nose/trachea/bronchus Produced in house or commercially supplied, e.g., MucilAir, human nasal, and EpiNasal, human nasal	MucilAir Yes [68] EpiNasal Yes (in house)	MucilAir 316 ± 31 [69] EpiNasal 110.7 ± 5.5 [70]	MucilAir Yes [69] EpiNasal Yes (in house)	MucilAir P-gp and BCRP [69] EpiNasal No studies	MucilAir Yes [71] EpiNasal Yes [70]	MucilAir Yes [72,73] EpiNasal Yes (in house)	MucilAir No studies EpiNasal Yes [70]
**RPMI-2650**	Human nose, squamous cell carcinoma, cancer cell line	Yes [66,74] No [75] Low [76]	75 [77] 66 ± 5 [66] 79.4 ± 5.2 [74] 55.1 ± 3.9 [78] 58 ± 5 [76]	Yes [78]	MRP1-5 Yes [79,80] P-gp and BCRP No [63] P-gp, MRP1, MRP2 and BCRP Yes [81] SLC transporters (PEPT2, OATP1A2, OATP4C1, OCT2, OCTN1 and OCTN2) [82]	Yes [63,74,77,81]	Yes [74,83,84]	Yes [85,86,87]
**UNCN3T**	Human bronchus, telomerase immortalized cell line	Yes [61,88]	229 ± 20 [61]	Yes [61]		Yes [61]	Yes [61]	No studies
**SPOC-1**	Rat trachea, spontaneously immortalized cell line	Yes [61,89]	217 ± 18 [61]			Yes [61]	Yes [61]	No studies
**Calu-3**	Human lung adenocarcinoma, cancer cell line	Yes * [61,66,76]	306 ± 53 [90] 368 ± 183 [61] 400 [91,92] 600 [93] 700–2500 [94] 624 ± 170 [76]	Yes [76,90]	PEPT1 [95] P-gp Yes [96,97] MRP1 Yes [92,96] MDR1 only in fully differentiated cells [92] BRCRP No [97] OCT1, OCT3 [98]	Yes [93,99]	Yes [83,100,101]	Yes [102,103]
**NuLi-1**	Human bronchus, transformed cell line	Yes [62] Serous phenotype [104]	~200 at 8 weeks ~450 at 6 weeks [104]	Yes [62,104]	No	Yes [62]	Yes [105,106]	No studies

ATP-binding cassette (ABC) transporters: P-gp—P glycoprotein; MDR1—Multidrug Resistance 1; MRP1—Multidrug Resistance-Associated Protein 1; MRP2—Multidrug Resistance-Associated Protein 2; BCRP—Breast Cancer Resistance Protein; SLC—solute carrier; PEPT—peptide transported; OCT—organic cation transporter; OATP—Organic Anion Transporter Polypeptide. * not in response to a physiological secretagogue and insufficient as a permeation barrier to testosterone [61].

**Table 3:** Differences in structure and architecture between the nasal cavities of human and widely used animal models [108].

	Sprague-Dawley Rat	Guinea Pig	Beagle Dog	Rhesus Monkey	Human
**Body Weight**	250 g	600 g	10 kg	7 kg	~70 kg
**Naris Cross-Section**	0.7 mm^2^	2.5 mm^2^	16.7 mm^2^	22.9 mm^2^	140 mm^2^
**Bend in Naris**	40°	40°	30°	30°	
**Length**	2.3 cm	3.4 cm	10 cm	5.3 cm	7–8 cm
**Greatest Vertical Diameter**	9.6 mm	12.8 mm	23 mm	27 mm	40–45 mm
**Surface Area (Both Sides of Nasal Cavity)**	10.4 cm^2^	27.4 cm^2^	220.7 cm^2^	61.6 cm^2^	181 cm^2^
**Volume (Both Sides)**	0.4 cm^3^	0.9 cm^3^	20 cm^3^	8 cm^3^	16–19 cm^3^ (does not include sinuses)
**Bend in Nasopharynx**	15°	30°	30°	80°	~90°
**Turbinate Complexity**	Complex scroll	Complex scroll	Very complex membranous	Simple scroll	Simple scroll

**Table 4:** Unmet needs in nasal biopharmaceutics: opportunities to improve the development of nasally administered drug products.

Biopharmaceutics Facet	Unmet Needs
Characterization	Can methods for assessing product quality be developed that account for how nasal products are actually used? Which biopharmaceutics aspects are not currently accounted for in Quality-by-Design-driven development of new nasal products? As innovative new models, e.g., nasal casts and dissolution methods, are introduced, when is standardization across models needed, and how is it achieved? How can methods for nasal dose collection be improved and standardized? Would a standard (compendial) nasal cast be useful for the field? How can particle size distributions be related quantitatively to the effectiveness and safety of a nasal product?
Physiologically based biopharmaceutics modeling	How can formulations be generated to test the parameters needed to build and validate deposition models? What deficiencies in our understanding of nasal delivery processes need to be addressed to develop mechanistic models? How can prediction accuracy be ascertained? What is needed to help scientists collaborate to bring together all the required elements for nasal delivery PBBM?
Nasal deposition	How can regional deposition be linked to downstream events, including (i) spray/wall interaction, liquid spreading and drip, as well as (ii) mucociliary clearance, dissolution and absorption? What in vitro methods are available for powders to understand and control (i) particle bounce and re-entrainment and (ii) the impact of coating surfaces? How should in vivo imaging studies be designed to validate these in vitro methods? What region(s) of the nose should be targeted for nasal delivery, i.e., is refinement in targeting needed to optimize therapy, e.g., for local delivery, systemic delivery, CNS diseases and vaccines?
Biological models	What regions and tissues/matrices of nasal cavity do we want to model? Is it necessary to recapitulate native tissue or just key (e.g., rate-limiting) features? How important is mucus in nasal drug delivery and how can mucus or mucus simulants be incorporated into test systems? How can existing/new models be validated to produce in vitro-in vivo correlations?
Non-clinical development	What is the link between regional deposition and effects? Are better non-clinical delivery devices being developed for smaller species? How can studies bridge across species, and are certain species more suited for different types of nasal drug delivery research question? Could a cross-species translation guidance (a “checklist”) be developed for animal-to-human scaling incorporating factors such as dose placement, particle size/velocity, dosing posture and breathing pattern? What gaps are there in understanding the relationship between administered dose and brain distributions? Can realistic expectations be defined for what can be achieved in non-clinical studies?
Clinical development	Which clinical nasal product development/evaluation methods are key to reverse engineering model development for prediction of in vivo performance in humans? What next steps should/could be taken to enhance our holistic understanding of the inter-relationship of the physicochemical properties of the drug, the dosage form and device performance characteristics on the robustness, rate and extent of drug deposition, residency, clearance and absorption for nasal products in humans?

## Data Availability

Not applicable.

## References

[1] Qayyum S.N., Ansari R.S., Ullah I., Siblini D. (2023). The FDA approves the second OTC naloxone—A step toward opioid crisis mitigation. Int. J. Surg..

[2] Borden T.J., Levien T.L., Baker D.E. (2022). Nasal Glucagon. Hosp. Pharm..

[3] Dworaczyk D.A., Hunt A.L., Di Spirito M., Lor M., Dretchen K.L., Lamson M.J., Pollock J., Ward T. (2024). A 13.2 mg epinephrine intranasal spray demonstrates comparable pharmacokinetics, pharmacodynamics, and safety to a 0.3 mg epinephrine autoinjector. J. Allergy Clin. Immunol. Glob..

[4] Kehagia E., Papakyriakopoulou P., Valsami G. (2023). Advances in intranasal vaccine delivery: A promising non-invasive route of immunization. Vaccine.

[5] Chen Y., Zhang C., Huang Y., Ma Y., Song Q., Chen H., Jiang G., Gao X. (2024). Intranasal drug delivery: The interaction between nanoparticles and the nose-to-brain pathway. Adv. Drug Deliv. Rev..

[6] Research and Markets Intranasal Drug and Vaccine Delivery Market Size, Share & Trends Analysis Report by Product, Dosage, Application, Distribution Channel, Region, and Segment Forecasts, 2025–2030. Research and Markets Reports October. https://www.researchandmarkets.com/reports/6024620/intranasal-drug-vaccine-delivery-market-size#src-pos-1.

[7] Grand View Research Nasal Drug Delivery Technology Market (2023–2030). https://www.grandviewresearch.com/industry-analysis/nasal-drug-delivery-technology-system-market#:~:text=The%20global%20nasal%20drug%20delivery,7.45%25%20from%202023%20to%202030.

[8] Research and Markets Intranasal Drug and Vaccine Delivery Market Size, Share & Trends Analysis Report by Product, Dosage, Application, Distribution Channel, Region, and Segment Forecasts, 2025–2030. Research and Markets Reports January. https://www.researchandmarkets.com/reports/516888/intranasal_drug_delivery_global_strategic?utm_source=BW&utm_medium=PressRelease&utm_code=j4zhdr&utm_campaign=1995813+-+Intranasal+Drug+Delivery+Research+Report+2024%3a+Nose-to-Brain+Drug+Delivery%3a+An+Evolving+Area+of+Interest&utm_exec=chdomspi.

[9] Scherließ R. (2020). Nasal formulations for drug administration and characterization of nasal preparations in drug delivery. Therapeutic Delivery.

[10] FDA Nasal Spray and Inhalation Solution, Suspension, and Spray Drug Products—Chemistry, Manufacturing, and Controls Documentation. https://www.fda.gov/regulatory-information/search-fda-guidance-documents/nasal-spray-and-inhalation-solution-suspension-and-spray-drug-products-chemistry-manufacturing-and.

[11] EMA Guideline on the Pharmaceutical Quality of Inhalation and Nasal Products. https://www.ema.europa.eu/en/documents/scientific-guideline/guideline-pharmaceutical-quality-inhalation-nasal-products_en.pdf.

[12] Bousquet J., Bachert C., Bernstein J., Canonica G.W., Carr W., Dahl R., Demoly P., Devillier P., Hellings P., Fokkens W. (2015). Advances in pharmacotherapy for the treatment of allergic rhinitis; MP29-02 (a novel formulation of azelastine hydrochloride and fluticasone propionate in an advanced delivery system) fills the gaps. Expert. Opin. Pharmacother..

[13] Schoenbrodt T., Egen M., Heyder K., Kohler D., Kranz Y., Mueller C., Schiewe J. (2010). Method Development for Deposition Studies in a Nasal Cast. Respir. Drug Deliv..

[14] Kundoor V., Dalby R.N. (2010). Assessment of nasal spray deposition pattern in a silicone human nose model using a color-based method. Pharm. Res..

[15] Chen J.Z., Kiaee M., Martin A.R., Finlay W.H. (2020). In vitro assessment of an idealized nose for nasal spray testing: Comparison with regional deposition in realistic nasal replicas. Int. J. Pharm..

[16] Williams G., Suman J.D. (2022). In Vitro Anatomical Models for Nasal Drug Delivery. Pharmaceutics.

[17] Kiaee M., Wachtel H., Noga M.L., Martin A.R., Finlay W.H. (2018). Regional deposition of nasal sprays in adults: A wide ranging computational study. Int. J. Numer. Methods Biomed. Eng..

[18] Calmet H., Inthavong K., Eguzkitza B., Lehmkuhl O., Houzeaux G., Vázquez M. (2019). Nasal sprayed particle deposition in a human nasal cavity under different inhalation conditions. PLoS ONE.

[19] Doub W.H., Suman J.M., Copley M., Goodey A.P., Hosseini S., Mitchell J.P. (2023). Laboratory Performance Testing of Aqueous Nasal Inhalation Products for Droplet/Particle Size Distribution: An Assessment from the International Pharmaceutical Aerosol Consortium on Regulation and Science (IPAC-RS). AAPS PharmSciTech.

[20] Baltz N., Scherließ R. Assessment of Mass Fraction Less Than 10 micron in Nasal Products–Method Considerations. Proceedings of the Drug Delivery to the Lungs.

[21] Baltz N., Svensson J., Skogevall M., Ohlsson A., Svensson M., Scherließ R. (2024). Advancing nasal formulation characterization: Considerations toward a robust and precise method to determine the mass fraction below 10 μm in nasal products. Aerosol Sci. Technol..

[22] Williams G., Blatchford C., Mitchell J.P. (2018). Evaluation of Nasal Inlet Ports Having Simplified Geometry for the Pharmacopeial Assessment of Mass Fraction of Dose Likely to Penetrate Beyond the Nasopharynx: A Preliminary Investigation. AAPS PharmSciTech.

[23] Baltz N., Scherließ R. Assessment of Nasal Products–Proposing a New Inlet. Proceedings of the Drug Delivery to the Lungs.

[24] Baltz N., Scherließ R. Fraction below 10 µm in Nasal Products–A Comparative Study of Laser Diffraction and Aerodynamic Assessment. Proceedings of the Drug Delivery to the Lungs.

[25] Anand O., Pepin X.J.H., Kolhatkar V., Seo P. (2022). The Use of Physiologically Based Pharmacokinetic Analyses—In Biopharmaceutics Applications -Regulatory and Industry Perspectives. Pharm. Res..

[26] Ding X., Kaminsky L.S. (2003). Human extrahepatic cytochromes P450: Function in xenobiotic metabolism and tissue-selective chemical toxicity in the respiratory and gastrointestinal tracts. Annu. Rev. Pharmacol. Toxicol..

[27] Oliveira P., Fortuna A., Alves G., Falcao A. (2016). Drug-metabolizing Enzymes and Efflux Transporters in Nasal Epithelium: Influence on the Bioavailability of Intranasally Administered Drugs. Curr. Drug Metab..

[28] Ohkubo K., Baraniuk J.N., Hohman R., Merida M., Hersh L.B., Kaliner M.A. (1998). Aminopeptidase activity in human nasal mucosa. J. Allergy Clin. Immunol..

[29] Gonda I. (1998). Mathematical modeling of deposition and disposition of drugs administered via the nose. Adv. Drug Deliv. Rev..

[30] Dave S., Kleinstreuer C., Chari S. (2022). An effective PBPK model predicting dissolved drug transfer from a representative nasal cavity to the blood stream. J. Aerosol Sci..

[31] U.S. Food and Drug Administration, Center for Drug Evaluation and Research (2024). FY 2024 GDUFA Science and Research Report. https://www.fda.gov/media/186225/download.

[32] Schroeter J.D., Tewksbury E.W., Wong B.A., Kimbell J.S. (2015). Experimental measurements and computational predictions of regional particle deposition in a sectional nasal model. J. Aerosol Med. Pulm. Drug Deliv..

[33] Keeler J.A., Patki A., Woodard C.R., Frank-Ito D.O. (2016). A Computational Study of Nasal Spray Deposition Pattern in Four Ethnic Groups. J. Aerosol Med. Pulm. Drug Deliv..

[34] Cabrera M., Michelet O., Piazzoni E., Erra B., Santiago-Ribeiro M.J., Maia S., Williams G., Vecellio L. (2017). Development of In Vitro Nasal Cast Imaging Techniques to Predict In Vivo Nasal Deposition. Respir. Drug Deliv. Eur..

[35] Manniello M.D., Hosseini S., Alfaifi A., Esmaeili A.R., Kolanjiyil A.V., Walenga R., Babiskin A., Sandell D., Mohammadi R., Schuman T. (2021). In vitro evaluation of regional nasal drug delivery using multiple anatomical nasal replicas of adult human subjects and two nasal sprays. Int. J. Pharm..

[36] Hosseini S., Alfaifi A., Esmaeili A.R., Edwards D., Schuman T., Longest W., Hindle M., Golshahi L. (2023). Effects of nasal anatomical characteristics and administration parameters on delivery of locally-acting drugs with suspension nasal sprays in adults. J. Aerosol Sci..

[37] Kimbell J.S., Segal R.A., Asgharian B., Wong B.A., Schroeter J.D., Southall J.P., Dickens C.J., Brace G., Miller F.J. (2007). Characterization of deposition from nasal spray devices using a computational fluid dynamics model of the human nasal passages. J. Aerosol Med..

[38] Inthavong K., Tian Z.F., Tu J.Y., Yang W., Xue C. (2008). Optimising nasal spray parameters for efficient drug delivery using computational fluid dynamics. Comput. Biol. Med..

[39] Rygg A., Hindle M., Longest P.W. (2016). Linking Suspension Nasal Spray Drug Deposition Patterns to Pharmacokinetic Profiles: A Proof-of-Concept Study Using Computational Fluid Dynamics. J. Pharm. Sci..

[40] Chen J., Finlay W.H., Vehring R., Martin A.R. (2024). Characterizing regional drug delivery within the nasal airways. Expert. Opin. Drug Deliv..

[41] Chen J., Martin A.R., Finlay W.H. (2021). Recent In Vitro and In Silico Advances in the Understanding of Intranasal Drug Delivery. Curr. Pharm. Des..

[42] Alfaifi A., Hosseini S., Esmaeili A.R., Walenga R., Babiskin A., Schuman T., Longest W., Hindle M., Golshahi L. (2022). Anatomically realistic nasal replicas capturing the range of nasal spray drug delivery in adults. Int. J. Pharm..

[43] Kiaee M., Wachtel H., Noga M.L., Martin A.R., Finlay W.H. (2019). An idealized geometry that mimics average nasal spray deposition in adults: A computational study. Comput. Biol. Med..

[44] Chen J.Z., Finlay W.H., Martin A. (2022). In Vitro Regional Deposition of Nasal Sprays in an Idealized Nasal Inlet: Comparison with In Vivo Gamma Scintigraphy. Pharm. Res..

[45] Duong K., Aisenstat M., Chen J.Z., Murphy B., Tavernini S., Wang H., Reiz B., Zheng J., Whittal R., McClary W.D. (2025). Characterization of Spray-Dried Powders Using a Coated Alberta Idealized Nasal Inlet. J. Aerosol Med. Pulm. Drug Deliv..

[46] Henriques P., Bicker J., Carona A., Miranda M., Vitorino C., Doktorovová S., Fortuna A. (2023). Amorphous nasal powder advanced performance: In vitro/ex vivo studies and correlation with in vivo pharmacokinetics. J. Pharm. Investig..

[47] Potts J.C., Penn L.C., Ahad J., Signorelli V., Jepras T.J., Mistry S.K., Mason L.M. Investigations into the Relationship Between Spray Dried Powder Particle Size and Deposition in Nose and Lung Analogues when Actuated from a Nasal Device. Proceedings of the Respiratory Drug Delivery.

[48] Mygind N., Dahl R. (1998). Anatomy, physiology and function of the nasal cavities in health and disease. Adv. Drug Deliv. Rev..

[49] Amini S.E., Gouyer V., Portal C., Gottrand F., Desseyn J.L. (2019). Muc5b is mainly expressed and sialylated in the nasal olfactory epithelium whereas Muc5ac is exclusively expressed and fucosylated in the nasal respiratory epithelium. Histochem. Cell Biol..

[50] Kennel C., Gould E.A., Larson E.D., Salcedo E., Vickery T., Restrepo D., Ramakrishnan V.R. (2019). Differential Expression of Mucins in Murine Olfactory Versus Respiratory Epithelium. Chem. Senses.

[51] Batts A.H., Marriott C., Martin G.P., Bond S.W., Greaves J.L., Wilson C.G. (1991). The use of a radiolabelled saccharin solution to monitor the effect of the preservatives thiomersal, benzalkonium chloride and EDTA on human nasal clearance. J. Pharm. Pharmacol..

[52] Rogers T.D., Button B., Kelada S.N.P., Ostrowski L.E., Livraghi-Butrico A., Gutay M.I., Esther C.R., Grubb B.R. (2022). Regional Differences in Mucociliary Clearance in the Upper and Lower Airways. Front. Physiol..

[53] McGhee E.O., Hart S.M., Urueña J.M., Sawyer W.G. (2019). Hydration Control of Gel-Adhesion and Muco-Adhesion. Langmuir.

[54] Ayoub M., Lethem M.I., Lansley A.B. (2021). The effect of ingredients commonly used in nasal and inhaled solutions on the secretion of mucus in vitro. Int. J. Pharm..

[55] Lock J.Y., Carlson T.L., Carrier R.L. (2018). Mucus models to evaluate the diffusion of drugs and particles. Adv. Drug Deliv. Rev..

[56] Lechanteur A., das Neves J., Sarmento B. (2018). The role of mucus in cell-based models used to screen mucosal drug delivery. Adv. Drug Deliv. Rev..

[57] Liu L., Tian C., Dong B., Xia M., Cai Y., Hu R., Chu X. (2021). Models to evaluate the barrier properties of mucus during drug diffusion. Int. J. Pharm..

[58] Mura S., Hillaireau H., Nicolas J., Kerdine-Römer S., Le Droumaguet B., Deloménie C., Nicolas V., Pallardy M., Tsapis N., Fattal E. (2011). Biodegradable Nanoparticles Meet the Bronchial Airway Barrier: How Surface Properties Affect Their Interaction with Mucus and Epithelial Cells. Biomacromolecules.

[59] Meindl C., Stranzinger S., Dzidic N., Salar-Behzadi S., Mohr S., Zimmer A., Fröhlich E. (2015). Permeation of Therapeutic Drugs in Different Formulations across the Airway Epithelium In Vitro. PLoS ONE.

[60] Cingolani E., Alqahtani S., Sadler R.C., Prime D., Stolnik S., Bosquillon C. (2019). In vitro investigation on the impact of airway mucus on drug dissolution and absorption at the air-epithelium interface in the lungs. Eur. J. Pharm. Biopharm..

[61] Lee D.F., Lethem M.I., Lansley A.B. (2021). A comparison of three mucus-secreting airway cell lines (Calu-3, SPOC1 and UNCN3T) for use as biopharmaceutical models of the nose and lung. Eur. J. Pharm. Biopharm..

[62] Sheikh Z., Bradbury P., Pozzoli M., Young P.M., Ong H.X., Traini D. (2020). An in vitro model for assessing drug transport in cystic fibrosis treatment: Characterisation of the CuFi-1 cell line. Eur. J. Pharm. Biopharm..

[63] Sibinovska N., Žakelj S., Kristan K. (2019). Suitability of RPMI 2650 cell models for nasal drug permeability prediction. Eur. J. Pharm. Biopharm..

[64] Economou E.C., Marinelli S., Smith M.C., Routt A.A., Kravets V.V., Chu H.W., Spendier K., Celinski Z.J. (2016). Magnetic Nanodrug Delivery Through the Mucus Layer of Air-Liquid Interface Cultured Primary Normal Human Tracheobronchial Epithelial Cells. Bionanoscience.

[65] Brinks V., Lipinska K., de Jager M., Beumer W., Button B., Livraghi-Butrico A., Henig N., Matthee B. (2019). The Cystic Fibrosis-Like Airway Surface Layer Is not a Significant Barrier for Delivery of Eluforsen to Airway Epithelial Cells. J. Aerosol Med. Pulm. Drug Deliv..

[66] Ladel S., Schlossbauer P., Flamm J., Luksch H., Mizaikoff B., Schindowski K. (2019). Improved In Vitro Model for Intranasal Mucosal Drug Delivery: Primary Olfactory and Respiratory Epithelial Cells Compared with the Permanent Nasal Cell Line RPMI 2650. Pharmaceutics.

[67] Mahallawi W.H., Aljeraisi T.M. (2021). In vitro cell culture model of human nasal-associated lymphoid tissue (NALT) to evaluate the humoral immune response to SARS-CoV-2 spike proteins. Saudi J. Biol. Sci..

[68] Welch J., Wallace J., Lansley A.B., Roper C. (2021). Evaluation of the toxicity of sodium dodecyl sulphate (SDS) in the MucilAir™ human airway model in vitro. Regul. Toxicol. Pharmacol..

[69] Mercier C., Jacqueroux E., He Z., Hodin S., Constant S., Perek N., Boudard D., Delavenne X. (2019). Pharmacological characterization of the 3D MucilAir™ nasal model. Eur. J. Pharm. Biopharm..

[70] Gadhave D.G., Quadros M., Ugale A.R., Goyal M., Ayehunie S., Gupta V. (2024). Mucoadhesive chitosan-poly (lactic-*co*-glycolic acid) nanoparticles for intranasal delivery of quetiapine—Development & characterization in physiologically relevant 3D tissue models. Int. J. Biol. Macromol..

[71] Furubayashi T., Inoue D., Nishiyama N., Tanaka A., Yutani R., Kimura S., Katsumi H., Yamamoto A., Sakane T. (2020). Comparison of Various Cell Lines and Three-Dimensional Mucociliary Tissue Model Systems to Estimate Drug Permeability Using an In Vitro Transport Study to Predict Nasal Drug Absorption in Rats. Pharmaceutics.

[72] Wallace J., Jackson G.R., Kaluzhny Y., Ayehunie S., Lansley A.B., Roper C., Hayden P.J. (2023). Evaluation of in vitro rat and human airway epithelial models for acute inhalation toxicity testing. Toxicol. Sci..

[73] Balogh Sivars K., Sivars U., Hornberg E., Zhang H., Brändén L., Bonfante R., Huang S., Constant S., Robinson I., Betts C.J. (2018). A 3D Human Airway Model Enables Prediction of Respiratory Toxicity of Inhaled Drugs In Vitro. Toxicol. Sci..

[74] Gonçalves V.S.S., Matias A.A., Poejo J., Serra A.T., Duarte C.M.M. (2016). Application of RPMI 2650 as a cell model to evaluate solid formulations for intranasal delivery of drugs. Int. J. Pharm..

[75] Berger J.T., Voynow J.A., Peters K.W., Rose M.C. (1999). Respiratory carcinoma cell lines. MUC genes and glycoconjugates. Am. J. Respir. Cell Mol. Biol..

[76] Martin J., Rittersberger R., Treitler S., Kopp P., Ibraimi A., Koslowski G., Sickinger M., Dabbars A., Schindowski K. (2024). Characterization of a primary cellular airway model for inhalative drug delivery in comparison with the established permanent cell lines CaLu3 and RPMI 2650. In Vitro Models.

[77] Wengst A., Reichl S. (2010). RPMI 2650 epithelial model and three-dimensional reconstructed human nasal mucosa as in vitro models for nasal permeation studies. Eur. J. Pharm. Biopharm..

[78] Chung T.W., Wu T.Y., Siah Z.Y., Liu D.Z. (2022). Antioxidative NAC-Loaded Silk Nanoparticles with Opening Mucosal Tight Junctions for Nasal Drug Delivery: An In Vitro and In Vivo Study. Pharmaceutics.

[79] Dolberg A., Reichl S. (2017). Activity of Multidrug Resistance-associated Proteins 1–5 (MRP1–5) in the RPMI 2650 Cell Line and Explants of Human Nasal Turbinate. Mol. Pharm..

[80] Dolberg A.M., Reichl S. (2016). Expression of P-glycoprotein in excised human nasal mucosa and optimized models of RPMI 2650 cells. Int. J. Pharm..

[81] Mercier C., Hodin S., He Z., Perek N., Delavenne X. (2018). Pharmacological Characterization of the RPMI 2650 Model as a Relevant Tool for Assessing the Permeability of Intranasal Drugs. Mol. Pharm..

[82] Dolberg A.M., Reichl S. (2018). Expression analysis of human solute carrier (SLC) family transporters in nasal mucosa and RPMI 2650 cells. Eur. J. Pharm. Sci..

[83] Stuetz H., Reihs E.I., Neuhaus W., Pflüger M., Hundsberger H., Ertl P., Resch C., Bauer G., Povoden G., Rothbauer M. (2023). The Cultivation Modality and Barrier Maturity Modulate the Toxicity of Industrial Zinc Oxide and Titanium Dioxide Nanoparticles on Nasal, Buccal, Bronchial, and Alveolar Mucosa Cell-Derived Barrier Models. Int. J. Mol. Sci..

[84] Wang H., Xu T., Han J., Zhang H., Hu S., Wei S., Cao M., Song Y., Yin D. (2025). Three-Dimensional Cultured Human Nasal Epithelial Cell Model for Testing Respiratory Toxicity and Neurotoxicity of Air Pollutants. Environ. Sci. Technol..

[85] Clementino A.R., Marchi C., Pozzoli M., Bernini F., Zimetti F., Sonvico F. (2021). Anti-Inflammatory Properties of Statin-Loaded Biodegradable Lecithin/Chitosan Nanoparticles: A Step Toward Nose-to-Brain Treatment of Neurodegenerative Diseases. Front. Pharmacol..

[86] Varga P., Németh A., Zeiringer S., Roblegg E., Budai-Szűcs M., Balla-Bartos C., Ambrus R. (2024). Formulation and investigation of differently charged β-cyclodextrin-based meloxicam potassium containing nasal powders. Eur. J. Pharm. Sci..

[87] Wong S., Brown A.D., Abrahams A.B., Nurzak A.N., Eltaher H.M., Sykes D.A., Veprintsev D.B., Fone K.C.F., Dixon J.E., King M.V. (2024). A Modified Cell-Penetrating Peptide Enhances Insulin and Oxytocin Delivery across an RPMI 2650 Nasal Epithelial Cell Barrier In Vitro. Pharmaceutics.

[88] Fulcher M.L., Gabriel S.E., Olsen J.C., Tatreau J.R., Gentzsch M., Livanos E., Saavedra M.T., Salmon P., Randell S.H. (2009). Novel human bronchial epithelial cell lines for cystic fibrosis research. Am. J. Physiol. Lung Cell Mol. Physiol..

[89] Randell S.H., Liu J.Y., Ferriola P.C., Kaartinen L., Doherty M.M., Davis C.W., Nettesheim P. (1996). Mucin production by SPOC1 cells--an immortalized rat tracheal epithelial cell line. Am. J. Respir. Cell Mol. Biol..

[90] Grainger C.I., Greenwell L.L., Lockley D.J., Martin G.P., Forbes B. (2006). Culture of Calu-3 cells at the air interface provides a representative model of the airway epithelial barrier. Pharm. Res..

[91] Furuse M., Itoh M., Hirase T., Nagafuchi A., Yonemura S., Tsukita S., Tsukita S. (1994). Direct association of occludin with ZO-1 and its possible involvement in the localization of occludin at tight junctions. J. Cell Biol..

[92] Rotoli B.M., Barilli A., Visigalli R., Ferrari F., Frati C., Lagrasta C.A., Lascia M.D., Riccardi B., Puccini P., Dall’Asta V. (2020). Characterization of ABC Transporters in EpiAirway™, a Cellular Model of Normal Human Bronchial Epithelium. Int. J. Mol. Sci..

[93] Foster K.A., Avery M.L., Yazdanian M., Audus K.L. (2000). Characterization of the Calu-3 cell line as a tool to screen pulmonary drug delivery. Int. J. Pharm..

[94] Loman S., Radl J., Jansen H.M., Out T.A., Lutter R. (1997). Vectorial transcytosis of dimeric IgA by the Calu-3 human lung epithelial cell line: Upregulation by IFN-gamma. Am. J. Physiol..

[95] Søndergaard H.B., Brodin B., Nielsen C.U. (2008). hPEPT1 is responsible for uptake and transport of Gly-Sar in the human bronchial airway epithelial cell-line Calu-3. Pflugers Arch..

[96] Hamilton K.O., Topp E., Makagiansar I., Siahaan T., Yazdanian M., Audus K.L. (2001). Multidrug resistance-associated protein-1 functional activity in Calu-3 cells. J. Pharmacol. Exp. Ther..

[97] Sibinovska N., Žakelj S., Roškar R., Kristan K. (2020). Suitability and functional characterization of two Calu-3 cell models for prediction of drug permeability across the airway epithelial barrier. Int. J. Pharm..

[98] Ingoglia F., Visigalli R., Rotoli B.M., Barilli A., Riccardi B., Puccini P., Dall’Asta V. (2015). Functional characterization of the organic cation transporters (OCTs) in human airway pulmonary epithelial cells. Biochim. Biophys. Acta.

[99] Mathia N.R., Timoszyk J., Stetsko P.I., Megill J.R., Smith R.L., Wall D.A. (2002). Permeability characteristics of calu-3 human bronchial epithelial cells: In vitro-in vivo correlation to predict lung absorption in rats. J. Drug Target..

[100] Jeong M.H., Han Y., Oh I.S., Kim D.M., Son D.W., Jung M.S., Yang H., Lee K., Shin J.Y., Kim H.R. (2021). Pre-validation of a Calu-3 epithelium cytotoxicity assay for predicting acute inhalation toxicity of chemicals. Toxicol. In Vitro.

[101] Jeong M.H., Kim H.R., Bang I.J., Yoo S.H., Lee S.J., Lee K.H., Chung K.H. (2019). In vitro model for predicting acute inhalation toxicity by using a Calu-3 epithelium cytotoxicity assay. J. Pharmacol. Toxicol. Methods.

[102] Florea B.I., Thanou M., Junginger H.E., Borchard G. (2006). Enhancement of bronchial octreotide absorption by chitosan and N-trimethyl chitosan shows linear in vitro/in vivo correlation. J. Control Release.

[103] Ghadiri M., Canney F., Pacciana C., Colombo G., Young P.M., Traini D. (2018). The use of fatty acids as absorption enhancer for pulmonary drug delivery. Int. J. Pharm..

[104] Molina S.A., Stauffer B., Moriarty H.K., Kim A.H., McCarty N.A., Koval M. (2015). Junctional abnormalities in human airway epithelial cells expressing F508del CFTR. Am. J. Physiol. Lung Cell Mol. Physiol..

[105] Feizi S., Awad M., Nepal R., Cooksley C.M., Psaltis A.J., Wormald P.J., Vreugde S. (2023). Deferiprone-gallium-protoporphyrin (IX): A promising treatment modality against Mycobacterium abscessus. Tuberculosis.

[106] Thomas N., Dong D., Richter K., Ramezanpour M., Vreugde S., Thierry B., Wormald P.-J., Prestidge C.A. (2015). Quatsomes for the treatment of Staphylococcus aureus biofilm. J. Mater. Chem. B.

[107] Harkema J.R., Carey S.A., Wagner J.G. (2006). The nose revisited: A brief review of the comparative structure, function, and toxicologic pathology of the nasal epithelium. Toxicol. Pathol..

[108] Reznik G., Stinson S.F. (1983). Experimental Nasal Carcinogenesis.

[109] Newman S.P., Illum L. (2004). Radionuclide imaging studies in the assessment of nasal drug delivery in humans. Am. J. Drug Deliv..

[110] Greiff L., Wollmer P., Erjefält I., Pipkorn U., Persson C.G. (1990). Clearance of 99mTc DTPA from guinea pig nasal, tracheobronchial, and bronchoalveolar airways. Thorax.

[111] Shah S.A., Berger R.L., McDermott J., Gupta P., Monteith D., Connor A., Lin W. (2015). Regional deposition of mometasone furoate nasal spray suspension in humans. Allergy Asthma Proc..

[112] Yamada K., Ishii K., Newman S. Optimization of Nasal Dosing Regimens for Olfactory Delivery. Proceedings of the Controlled Release Society (CRS).

[113] Thwaites R.S., Ito K., Chingono J.M.S., Coates M., Jarvis H.C., Tunstall T., Anderson-Dring L., Cass L., Rapeport G., Openshaw P.J. (2017). Nasosorption as a Minimally Invasive Sampling Procedure: Mucosal Viral Load and Inflammation in Primary RSV Bronchiolitis. J. Infect. Dis..

[114] Bäckman P., Arora S., Couet W., Forbes B., de Kruijf W., Paudel A. (2018). Advances in experimental and mechanistic computational models to understand pulmonary exposure to inhaled drugs. Eur. J. Pharm. Sci..

[115] Bäckman P., Cabal A., Clark A., Ehrhardt C., Forbes B., Hastedt J., Hickey A., Hochhaus G., Jiang W., Kassinos S. (2022). iBCS: 2. Mechanistic Modeling of Pulmonary Availability of Inhaled Drugs versus Critical Product Attributes. Mol. Pharm..

[116] Forbes B., Bäckman P., Cabal A., Clark A., Ehrhardt C., Hastedt J.E., Hickey A.J., Hochhaus G., Jiang W., Kassinos S. (2025). iBCS: 4. Application of the Inhalation Biopharmaceutics Classification System to the Development of Orally Inhaled Drug Products. Mol. Pharm..

